# The Fleas (Siphonaptera) in Iran: Diversity, Host Range, and Medical Importance

**DOI:** 10.1371/journal.pntd.0005260

**Published:** 2017-01-09

**Authors:** Naseh Maleki-Ravasan, Samaneh Solhjouy-Fard, Jean-Claude Beaucournu, Anne Laudisoit, Ehsan Mostafavi

**Affiliations:** 1 Malaria and Vector Research Group, Biotechnology Research Center, Pasteur Institute of Iran, Tehran, Iran; 2 Research Centre for Emerging and Reemerging infectious diseases, Pasteur Institute of Iran, Akanlu, Kabudar Ahang, Hamadan, Iran; 3 Department of Epidemiology and Biostatistics, Pasteur institute of Iran, Tehran, Iran; 4 University of Rennes, France Faculty of Medicine, and Western Insitute of Parasitology, Rennes, France; 5 Evolutionary Biology group, University of Antwerp, Antwerp, Belgium; 6 School of Biological Sciences, University of Liverpool, Liverpool, United Kingdom; 7 CIFOR, Jalan Cifor, Situ Gede, Sindang Barang, Bogor Bar., Jawa Barat, Indonesia; University of Tennessee, UNITED STATES

## Abstract

**Background:**

Flea-borne diseases have a wide distribution in the world. Studies on the identity, abundance, distribution and seasonality of the potential vectors of pathogenic agents (e.g. *Yersinia pestis*, *Francisella tularensis*, and *Rickettsia felis*) are necessary tools for controlling and preventing such diseases outbreaks. The improvements of diagnostic tools are partly responsible for an easier detection of otherwise unnoticed agents in the ectoparasitic fauna and as such a good taxonomical knowledge of the potential vectors is crucial. The aims of this study were to make an exhaustive inventory of the literature on the fleas (Siphonaptera) and range of associated hosts in Iran, present their known distribution, and discuss their medical importance.

**Methodology/Principal Findings:**

The data were obtained by an extensive literature review related to medically significant fleas in Iran published before 31^st^ August 2016. The flea-host specificity was then determined using a family and subfamily-oriented criteria to further realize and quantify the shared and exclusive vertebrate hosts of fleas among Iran fleas. The locations sampled and reported in the literature were primarily from human habitation, livestock farms, poultry, and rodents’ burrows of the 31 provinces of the country. The flea fauna were dominated by seven families, namely the Ceratophyllidae, Leptopsyllidae, Pulicidae, Ctenophthalmidae, Coptopsyllidae, Ischnopsyllidae and Vermipsyllidae. The hosts associated with Iran fleas ranged from the small and large mammals to the birds. Pulicidae were associated with 73% (56/77) of identified host species. Flea-host association analysis indicates that rodents are the common hosts of 5 flea families but some sampling bias results in the reduced number of bird host sampled. Analyses of flea-host relationships at the subfamily level showed that most vertebrates hosted fleas belgonging to 3 subfamilies namely Xenopsyllinae (n = 43), Ctenophthalminae (n = 20) and Amphipsyllinae (n = 17). *Meriones persicus* was infested by 11 flea subfamilies in the arid, rocky, mountainous regions and Xenopsyllinae were hosted by at least 43 mammal species. These findings place the Persian jird (*M*. *persicus*) and the Xenopsyllinae as the major vertebrate and vector hosts of flea-borne diseases in Iran including *Yersinia pestis*, the etiological agent of plague. We found records of at least seven vector-borne pathogenic agents that can potentially be transmitted by the 117 flea species (or subspecies) of Iran.

**Conclusions/Significance:**

Herein, we performed a thorough inventary of the flea species and their associated hosts, their medical importance and geographic distribution throughout Iran. This exercise allowed assessing the diversity of flea species with the potential flea-borne agents transmission risk in the country by arranging published data on flea-host associations. This information is a first step for issuing public health policies and rodent-flea control campaigns in Iran as well as those interested in the ecology/epidemiology of flea-borne disease.

## Author Summary

The data about flea-borne emerging or re-emerging infections throughout Iran are limited. This paper showed that the flea fauna of Iran were dominated by seven families. Moreover flea-host association analysis indicates that rodents are common hosts of flea families and most vertebrates hosted fleas belonging to the subfamilies Xenopsyllinae, Ctenophthalminae and Amphipsyllinae. We showed that the Persian jird (*Merions persicus* Blanford, 1875) and the Xenopsyllinae are respectively the major vertebrate and potential vectors of flea-borne diseases in Iran. Further efforts are needed to inventorize and screen molecularly wild and domestic mammals flea fauna (>3kg) in order to monitor the risk of and control flea-borne infections in Iran, especially in the ecoregions with high diversity of flea and host species and in the old endemic plague foci of the country.

## Introduction

Vector-borne diseases (VBDs) are globally responsible for more than 17% of all infectious diseases [1]. There are a large number of viral, rickettsial, bacterial and parasitic diseases that are transmitted by insect vectors [2]. In the last two decades, many zoonotic VBDs have emerged in areas where they previously did not occur, and the incidence of these diseases both in endemic areas and outside their known range has increased [3]. In recent years, most studies on zoonotic diseases have focused on tick- and mosquito-borne diseases, less attention has been given to flea-borne diseases[4].

Fleas (Siphonaptera) are small, bloodsucking or hematophagous ectoparasites that may transmit pathogens through several possible mechanisms, including: contaminated feces (e.g. *R*. *typhi*, *B*. *henselae*), soiled mouthparts (e.g. *Y*. *pestis*, viral pathogens), regurgitation of gut contents (e.g. *Y*. *pestis*), and infectious saliva (e.g. *R*. *felis* in salivary glands)[4].

Over 2500 flea species belonging to 16 families and 238 genera have been described worldwide [5]. Fleas are mainly ectoparasites of mammals while birds are infested by only 6% of the known species. This is partly due to reduced collection efforts and sampling bias as only few bird fleas are in close contact with humans [6]. Fleas are one of the most common insect groups that can serve as vector and intermediate host of pathogenic zoonotic agents between vertebrate hosts, including humans [4, 7–8]. Fleas can have a direct pathogenic effect by causing allergic dermatitis [9–10] or paralysis subsequent to the injection of saliva into their hosts skin or blood [11]. Notorious human pathogens such as *Yersinia pestis* (plague), *Rickettsia typhi* (murine typhus), *Francisella tularensis* (tularemia) and *Bartonella henselae* (cat scratch disease) are transmitted by fleas [12–15].

Some fleas tend to be host specific (restricted or specialist), but others have a wide host range (permissive, opportunistic). The permissive species group are more significant than the restricted ones, because they can spread infectious agents among and within their multiple hosts and across a diverse series of habitats [6]. In order to prevent or control the occurrence and spread of flea-borne diseases, it is thus necessary to establish a taxonomical inventory of the flea fauna and their specific distribution range.

Climate changes, due to global warming and human intervention, have led to changes in the biological parameters and distribution ranges of vectors and hence of VBDs [16]. On the bases of vulnerability assessments and models, it is predicted that climate change will result in raised incidence of communicable diseases embracing VBDs; however the short and long term effects will be mitigated and will be linked to vector life cycles (e.g.: developments of preimaginal stages) and geographic area [17]. Reasonable proofs tend to suggest that changes in climatic factors may affect VBDs incidence especially acting on the off-host developmental life stages of arthropods and hence disease transmission dynamics. Insects as poikilotherm organisms have no internal control of their body temperatures, and as such depend on their host(s)—the imago as a transient habitat -, and abiotic conditions for survival, which both condition their vector capacity, as well as their reproduction rate[18]. Moreover, vector capacity is linked to the nature of the pathogen transmitted, survival rate inside its vector host—which may or may not affect vector fitness—and incubation or turnover rate that is inversely proportional to temperature[19]. Moreover, climate and human behavior changes increase human exposures to vectors and the pathogenic agents they transmit [20]. Studies of plague transmission in the U.S.A, China and Kazakhstan have found that the patterns of human or rodent plague are shifting as temperatures warms up or link to climatic oscillations (such as El Niño) and precipitation pattern [20].

Iranian physicians were familiar with the human plague for a long time. Although there are little information about the situation of plague from earlier centuries, more documented evidence are available from the 19th and 20th centuries. As a matter of fact, faunistic studies of Iranian fleas have been carried out mainly about 60 years ago in a context of plague research and most species described at the time were collected and described off plague hosts [21]. When plague research stopped, flea inventories did so too and there are no current updates on the flea fauna of Iran. However, a recent study detected antibodies against *Y*. *pestis* in dogs—known to be a good sentinels for plague surveillance- while human plague hasn’t been reported for 50 years [22]. This finding triggered some concern about the possible plague reemergence in the countryside, in the old plague foci and called for an update on the state-of-knowledge of the flea diversity in the country. The aims of the present study were to update by reviewing the current state of knowledge of the Iranian Siphonaptera diversity, their host range and especially the medically important species.

## Methods

This review was based on a search of the online scientific databases (Scientific Information Database) PubMed and Google Scholar from 1952 through 31^st^ August 2016. Keywords—submitted in English, French, Turkish and Russian—for the search were “flea AND fauna AND Iran”; “Iran AND puce”, “Iran AND siphonaptera”; “Iran AND ectoparasite”. Searches were conducted in the titles, abstracts, keywords and full text. The majority of our knowledge on the Siphonaptera of Iran is derived from plague studies[23], the concept of “telluric plague” is coeval with these researches[24] and studies of two flea specialists, the Iranian Farhang-Azad and the French J.M. Klein.

In each case the flea species, its host, and location of sampling were extracted from the published papers. The flea distribution maps were prepared using ArcGIS (ArcGIS version 9.3, ESRI). An online software were used to further classify and quantify the shared and exclusive vertebrate hosts of fleas with the “family or subfamily” filtering criteria[25].

## Results

### Literature review

**Fig 1:**
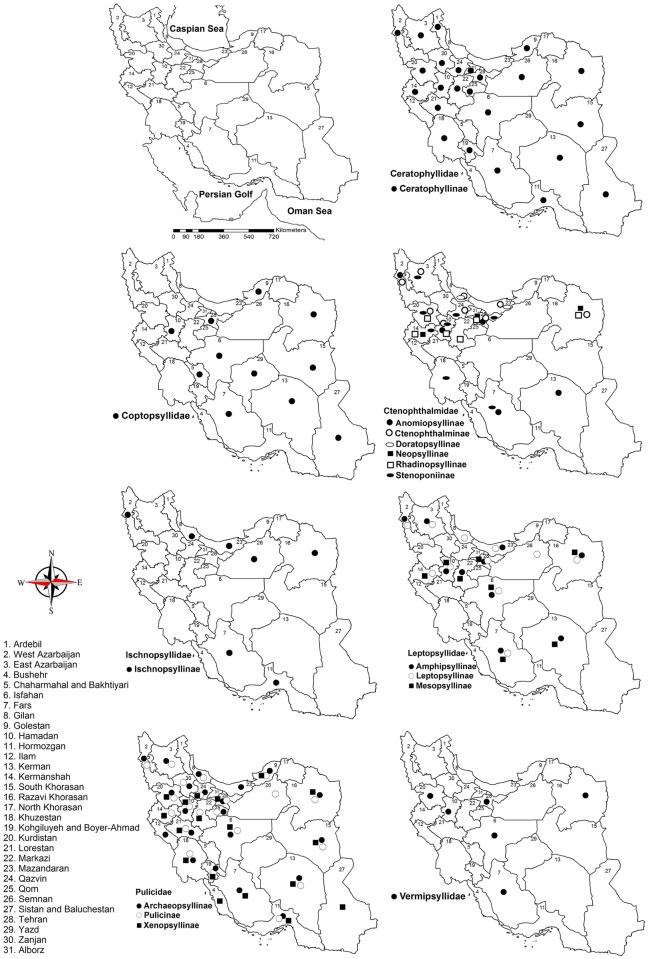
Distribution maps of studied fleas (sub-) family in Iran.

The data for this study were extracted from about 100 relevant papers in English, French, Istanbul Turkish or Russian. Faunistic reviews of the medically significant fleas showed the presence of fleas through 31 Iranian provinces (Fig 1). In the old classification of Iran provinces used by Farhang-Azad (1972b), the Khorasan province, which was the largest province of Iran in the plague research era, is currently divided in three provinces namely Razavi Khorasan, North Khorasan, and South Khorasan. This means that the spatial scale of the flea range resolution is less accurate in the old literature as it covers a larger area where the flea and their host are not homogenously found. Based on the information in the studied papers, the sampling locations mainly were human houses, animal husbandry premises, poultry farms, and rodents’ burrows.

### Flea diversity

According to the literature, about 117 species or subspecies of fleas belonging to 7 families and 35 genera have been described in Iran. Most flea species reported in the studied literature belonged to the Ceratophyllidae (n = 33), Leptopsyllidae (n = 24), Pulicidae (n = 21), Ctenophthalmidae (n = 20) and Coptopsyllidae (n = 9) families. The flea species of the Ischnopsyllidae (bat-fleas) and Vermipsyllidae (carnivore-fleas) families consisted of only 6 and 4 species of the whole collection respectively (Tables 1 and 2).

**Table 1:** Studied species, places of sampling and hosts of studied fleas.

Family	Subfamily	Fleas species	Associated host	Province (Locality)	Ref.
Ceratophyllidae	Ceratophyllinae	*Callopsylla aff*. *Caspia*	Lag: *Ochotona rufescens*	Razavi Khorasan (Mashhad)	[26]
		*C*. *caspia caspia*	Rod.: *Microtus arvalis*	Alborz Mountains	[27–28]
		*C*. *saxatilis*	Rod.: *Microtus nivalis*	East Azarbaijan (Tabriz)	[26–28]
		*C*. *tiflovi*	Rod.: *Citellus fulvus*; Lag.: *Ochotona rufescens*	Isfahan, Tehran (Daleh Tani), Semnan (Shahrood), South Khorasan (Tabas), Razavi Khorasan (Mashhad, Asi Bolagh Ghoochan), Golestan (Shahkooh)	[21, 26]
		*Ceratophyllus fringillae*	Bird*s*: *Motacilla alba*, *Galerida cristata*, *Passer domesticus*	Isfahan (Oshtor-Jan, Ali Abad)	[26, 29]
		*C*. *gallinae*	Birds: *Gallus gallus*, *Motacilla alba*, *Passer domesticus*	Fars, Qazvin, Qom, Hormozgan, Isfahan (Ali Abad), Kerman, South Khorasan, Razavi Khorasan, Lorestan (Khorram-Abad), Kohgiluyeh and Boyer-Ahmad, Kurdistan, Tehran, Zanjan	[26, 29–30]
		*C*. *spinosus*	Carn.: *Vulpes vulpes* (acc., a bird’s flea)	Hamadan (Agh Bolagh Morshed)	[21, 26]
		*Citellophilus trispinus*	Rod.: *Citellus fulvus*	Tehran, Razavi Khorasan (Mashhad, Sabzevar, Asi Bolagh, Ghoochan, Lotf Abad, Akhlamad), South Khorasan (Tabas)	[21, 26]
		*Myxopsylla jordani*	Rod.: *Dryomys nitedula*	Razavi Khorasan (Mashhad)	[26–27]
		*Nosopsyllus baltazardi*	Rod.: *Gerbillus nanus*, *G*. *cheesmani*, *Tatera indica*, *Meriones persicus*, *M*. *crassus*, *M*. *libycus*, *Rhombomys opimus*	Tehran, Fars (Shiraz), Kerman	[26]
		*N*. *consimilis*	Rod.: *Microtus socialis*	Ardebil (Moghan)	[31]
		*N*. *farahae*	Rod.: *Microtus socialis*	West Azarbaijan (Urmia)	[31]
		*N*. *fasciatus*	Rod.: *Rattus rattus*, *R*. *norvegicus*, *Mus musculus*, *Cricetulus migratorius*, *Nesokia indica*, *Allactaga williamsi*, *Mesocricetus auratus*	Lorestan (Khorram-Abad, Weysian, Chaghalvand, Papi, Chegini, Zagheh), Tehran, Gilan (Rasht)	[21, 26, 32]
		*N*. *fidus*	Rod.: *Gerbillus nanus*	Sistan and Baluchestan (Zabol)	[31]
		*N*. *iranus attenuates*	Rod.: *Meriones tristrami*	Kurdistan	[27]
		*N*. *iranus iranus*	Rod.: *Meriones persicus*, *M*. *libycus*, *M*. *vinogradovi*, *M*. *tristrami*, *Rattus norvegicus*, *Microtus irani*, *M*. *socialis*, *Cricetulus migratorius*; Insect.: *Hemiechinus auritus* (acc.); Carn.: *Vulpes vulpes* (acc.)	Kurdistan, Tehran, Qazvin, Hamadan (Agh Bolagh Morshed, Akanlu), Lorestan (Khorram-Abad, Weysian, Chaghalvand, Papi, Chegeni, Zagheh), East Azarbaijan (Tabriz), West Azarbaijan (Urmia), Kermanshah, Ardebil (Bilesauvar)	[21, 26, 33–34]
		*N*. *iranus theodori*	NS	NS	[27]
		*N*. *laeviceps gorganus*	Rod.: *Rhombomys opimus*, *Meriones libycus*, *M*. *persicus*, *Microtus socialis*	Golestan (Dash Boroon)	[21, 26]
		*N*. *londiniensis londiniensis*	Rod.: *Mus musculus*	NS	[27]
		*N*. *medus*	Rod.: *Mus musculus;* Insect: *Crocidura russula*, *C*. *leucodon*	Kermanshah, Hamadan (Akanlu), West Azarbaijan (Urmia)	[21, 26, 35]
		*N*. *mikulini* (= *N*. *parsus*)	Rod.: *Mus musculus*, *Rattus norvegicus*, *Cricetulus migratorius*, *Microtus irani*, *M*. *arvalis*, *M*. *socialis*, *Mesocricetus auratus*, *Meriones persicus*, *M*. *libycus*, *M*. *vinogradovi*; Lag.: *Ochotona rufescens*, *Lepus capensis*; Carn.: *Vulpes vulpes*	Hamadan (Akanlu), Tehran (Daleh Tani), Kurdistan	[21, 26, 36]
		*N*. *mokrzecki*	Rod.: *Cricetulus migratorius*, *Microtus socialis*	East Azarbaijan (Tabriz)	[26]
		*N*. *monstrosus vlasovi*	Rod.: *Rhombomys opimus*	Razavi Khorasan (Lotf Abad)	[37]
		*N*. *philipovi rashti*	Rod.: *Mus musculus*, *Calomyscus bailwardi*, *Ellobius fuscocapillus*, *Microtus arvalis*, *M*. *socialis*	Tehran, Razavi Khorasan (Mashhad)	[26]
		*N*. *pringlei*	Rod.: *Tatera indica*, *Rhombomys opimus*, *Jaculus jaculus*, *J*. *blanfordi*, *Meriones crassus*, *M*. *libycus*, *M*. *persicus*, *Gerbillus nanus*	Isfahan (Yeklengy), Tehran (Seid Abad, Kan), Khuzestan (Shoosh). Markazi (Aziz Abad, Mahallat), Isfahan (Ghaleh Tappe)	[21, 26, 29]
		*N*. *sarinus aryanus*	Rod.: *Rattus norvegicus*	Khuzestan (Abadan)	[21, 26]
		*N*. *sarinus parthius*	Rod.: *Mus musculus*, *Meriones persicus*	Kerman, Fars (Shiraz)	[21, 26, 33]
		*N*. *sarinus sarinus*	Rod.: *Mus musculus*	NS	[27, 37]
		*N*. *tersus tarsus*	Rod.: *Rhombomys opimus*	NS	[26–27]
		*N*. *turkmenicus turkmenicus*	Rod.: *Rhombomys opimus*, *Gerbillus nanus*, *Cricetulus migratorius*, *Meriones persicus*, *M*. *libycus*	Razavi Khorasan (Sabzehvar, Ghoochan, Lotf Abad)	[26]
		*N*. *vlasovi*	Rod.: *Rhombomys opimus*, *Meriones meridianus*, *M*. *crassus*, *M*. *libycus*	Fars (Shiraz), Kerman	[26]
		*N*. *ziarus*	Rod.: *Rhombomys opimus*, *Meriones persicus*, *M*. *libycus*, *Jaculus blanfordi*	Isfahan	[21, 29]
		*Paraceras melis melis*	Carn.: *Meles meles*	Hamadan (Agh Bolagh Morshed, Akanlu, Ghare Dagh, Gueurach)	[21, 26]
Coptopsyllidae	NS	*Coptopsylla bairamaliensis*	Rod.: *Rhombomys opimus*, *Meriones persicus*	Razavi Khorasan (Hossain Abad, Loft Abad)	[26, 38–40]
		*C*. *iranica*	Rod.: *Gerbillus nanus*, *Meriones libycus*, *M*. *persicus*, *M*. *meridianus*, *M*. *crassus*	Golestan (Dash-Boroun), Isfahan, Razavi Khorasan (Lotf Abad), Tehran, Sistan and Baluchestan (Bampour, Zahedan), Yazd, South Khorasan (Tabas)	[38–39]
		*C*. *joannae*	NS	NS	[27]
		*C*. *lamellifer dubinini*	Rod.: *Rhombomys opimus*, *Meriones vinogradovi*, *M*. *libycus*, *M*. *persicus*; Carn.: *Meles meles* (acc.)	Golestan (DashBoroun), Razavi Khorasan (Loft Abad, Shandiz), Hamadan (Akanlu), Isfahan (Dorche Piaz, Yeklengy)	[21, 26, 29, 38–39]
		*C*. *lamellifer lamellifer*	Rod.: *Rhombomys opimus*, *Meriones persicus*, *M*. *vinogradovi*, *M*. *libycus*; Carn.: *Meles meles* (acc.).	Golestan (Torkman Sahra), Razavi Khorasan (Lotf Abad, Shandiz), Isfahan (Dorcheh)	[26, 38, 40]
		*C*. *lamellifer rostrata*	*Rhombomys opimus*	Chaharmahal and Bakhtiyari, Isfahan (Yeklengy, Dorche Piaz)	[26, 38, 41]
		*C*. *mesghalii*	*Rhombomys opimus*	Chaharmahal and Bakhtiyari, Isfahan (Yeklengy, Dorche Piaz)	[26, 29, 38–40]
		*C*. *mofidii*	Rod.: *Tatera indica*, *Gerbillus nanus*, *Meriones libycus*, *M*. *crassus*, *M*. *persicus*, *Rhombomys opimus*, *Calomyscus bailwardi*, *Cricetulus migratorius*	Fars (Fasa to Jahrom), Golestan (DashBoroun), Isfahan, Razavi Khorasan (Lotf Abad), Tehran, Kerman (Fahraj, Hossein Abad), Yazd (Taft), South Khorasan (Tabas)	[26, 38–39]
		*C*. *neronovi*	Rod.: *Meriones persicus*, *M*. *crassus*	Sistan and Baluchestan	[38]
Ctenophthalmidae	Anomiopsyllinae	*Wagnerina schelkovnikovi*	Rod.: *Mus musculus*, *Calomyscus bailwardi*, *Cricetulus migratorius*, *Meriones persicus*, *Mesocricetus auratus*, *Nesokia indica*	Tehran (Firooz Kooh, Ghale Morghi), Hamadan (Akanlu), West Azarbaijan (Urmia), Fars (Shiraz), Kerman	[21, 26]
	Ctenophthalminae	*Ctenophthalmus angulosus*	Insect: *Crocidura russula*; Rod.: *Pitymys majori*	Mazandaran (Veysar), Ghilan (Assalem)	[37]
		*C*. *congener congener*	Insect: *Talpa europaea;* Rod.: *Apodemus sylvaticus*, *Meriones persicus*, *Microtus arvalis*, *M*. *socialis*	NS	[42]
		*C*. *congener nadimi*	Rod.: *Microtus arvalis*	Razavi Khorasan (Mashhad)	[26–27, 43]
		*C*. *dolichus bair*	NS	Razavi Khorasan (Mashhad)	[26]
		*C*. *dolichus kurdensis*	Rod.: *Cricetulus migratorius*, *Meriones persicus*, *M*. *libycus*, *M*. *vinogradovi*	Kurdistan, Tehran	[21, 26, 44]
		*C*. *iranus persicus*	Rod.: *Mesocricetus auratus*, *Cricetulus migratorius*, *Nesokia indica*, *Meriones persicus*, *M*. *libycus*, *M*. *vinogradovi*, *M*. *tristrami*, *Microtus irani*, *Mus musculus*; Carn.: *Vulpes vulpes* (acc.)	Hamadan (Akanlu), Qazvin, East Azarbaijan (Tabriz)	[21, 26, 33]
		*C*. *lewisi*	Rod.: *Pitymys majori*	Mazandaran (Dasht-Lateh)	[37]
		*C*. *proximus*	Insect: *Crocidura russula*, *C*. *leucodon*; Rod.: *Apodemus sylvaticus*, *A*. *mystacinus*	Mazandaran (Veysar, Dasht-Lateh), Ghilan (Assalem), West Azarbaijan (Urmia)	[37]
		*C*. *rettigi smiti*	Rod.: *Mesocricetus auratus*, *Allactaga williamsi*	Hamadan (Agh Bolagh Morshed)	[21, 26, 36]
		*Palaeopsylla copidophora*	Insect: *Talpa caeca*	Ghilan (Assalem)	[37]
	Doratopsyllinae	*Doratopsylla dampfi irana*	Insect: *Sorex minutus*, *Crocidura russula*; Rod.: *Apodemus mystacinus*	Mazandaran (Veysar), Ghilan (Assalem)	[37]
	Neopsyllinae	*Neopsylla pleskei ariana*	Rod.: *Citellus fulvus*, *Meriones persicus*	Tehran (FiroozKooh), Razavi Khorasan (Mashhad)	[21, 26, 28, 45]
		*N*. *setosa setosa*	Rod.: *Citellus fulvus*, *Cricetulus migratorius*	Razavi Khorasan (Asi Bolagh Ghoochan)	[26]
		*N*. *teratura rhagesa*	Rod.: *Cricetulus migratorius*	Tehran, Kermanshah	[21, 26, 44]
	Rhadinopsyllinae	*Rhadinopsylla bivirgis*	Rod.: *Meriones libycus*, *M*. *persicus*	Razavi Khorasan (Asi Bolagh Ghoochan)	[26]
		*R*. *syriaca*	Rod.: *Meriones libycus*	Markazi (Aziz abad), Tehran, Kermanshah	[21, 26]
		*R*. *ucrainica*	Rod.: *Meriones persicus*, *M*. *tristrami*, *M*. *libycus*, *M*. *vinogradovi*, *Mesocricetus auratus*, *Microtus irani*,*M*. *socialis*, *Cricetulus migratorius*, *Mus musculus*	Tehran (Roodehen, Ghaleh Morghi, Seid Abad), Kurdistan, Hamadan (Agh Bolagh Morshed, Akanlu)	[21, 26]
	Stenoponiinae	*Stenoponia tripectinata irakana* (*= S*. *insperata irakana*)	Rod.: *Gerbillus nanus*, *G*. *dasyurus*, *Meriones persicus*, *M*. *libycus*, *M*. *vinogradovi*, *M*. *tristrami*, *M*. *crassus*, *Calomyscus bailwardi*, *Tatera indica Rhombomys opimus*, *Citellus fulvus*; Carn.: *Meles meles*	Khuzestan (Shoosh), Qazvin, Kurdistan, Hamadan (Akanlu) Tehran, East Azarbaijan (Tabriz), Kermanshah, Fars (Shiraz)	[21, 26, 34, 46]
		*S*. *vlasovi*	Rod.: *Rhombomys opimus*, *Meriones persicus*	Isfahan (Yeklengy), Razavi Khorasan (Mashhad)	[26, 29]
Ischnopsyllidae	Ischnopsyllinae	*Chiropteropsylla brockmani*	Chi.: *Aselia tridens*	Hormozgan (Roodan, Minab), Fars (Shiraz)	[26, 43]
		*Ischnopsyllus dolosus*	Chi.: *Myotis blythi*, *Pipistrellus pipistrellus*	Azarbaijan	[47]
		*I*. *elongates*	Chi.: *Aselia tridens* (acc.), *Nyctalus noctula*	Mazandaran (Tonekabon)	[47]
		*I*. *octactenus*	Chi.: *Pipistrellus pipistrellus*, *Pipistrellus kuhlii*	Ramsar, Ghilan (Rasht), Razavi Khorasan (Mashhad)	[21, 26, 37, 43, 48]
		*I*. *petropolitanus*	Chi.: *Plecotus macrobullaris*	Semnan (Gandab)	[47]
		*Rhinolophopsylla unipectinata*	Chi.: *Rhinolophus blasii*, *Pipistrellus pipistrellus* (acc.), *Aselia tridens* (acc.).	Razavi Khorasan (Mashhad)	[26, 47]
Leptopsyllidae	Amphipsyllinae	*Amphipsylla argoi*	Rod.: *Calomyscus bailwardi*	Isfahan (Ghaleh Tappeh)	[21, 26]
		*A*. *parthiana*	Rod.: *Microtus socialis*, *M*. *arvalis*	Razavi Khorasan (Mashhad)	[26–28]
		*A*. *rossica rossica*	Rod.: *Microtus irani*, *М*. *Meles meles*	Hamedan (Agh BolaghMorshed)	[21, 26]
		*A*. *schelkovnikovi schelkovnikovi* (*= A*. *s*. *irana*)	Rod.: *Meriones persicus*, *M*. *libycus*, *M*. *vinogradovi*, *Mus musculus*, *Cricetulus migratorius*, *Rattus norvegicus*, *Microtus irani*, *Mesocricetus auratus*; Carn.: *Vulpes vulpes* (acc.).	Hamadan (Agh Bolagh Morshed, Akanlu), Tehran (Firoozkooh), Isfahan (Ghaleh Tappeh), Razavi Khorasan (Mashhad)	[21, 26, 28]
		*Ctenophyllus rufescens*	Lag.: *Ochotona rufescens*; Rod.: *Calomyscus bailwardi*	Markazi (Mahallat)	[21, 26, 28]
		*Frontopsylla ambigua*	Rod.: *Apodemus mystacinus*, *Mus musculus*	Razavi Khorasan (Mashhad)	[26]
		*Ophthalmopsylla volgensis arnoldi*	Rod.: *Allactaga elater*, *A*. *williamsi*, *Cricetulus migratorius*, *Meriones persicus*, *M*. *libycus*, *M*. *vinogradovi*; Insect.: *Hemiechinus auritus* (acc.).	Hamadan (Agh Bolagh Morshed, Akanlu) Tehran, East Azarbaijan (Tabriz)	[21, 26]
		*O*. *volgensis impersia*	Rod.: *Allactaga elater*	Mazandaran (Sari)	[26, 49]
		*O*. *volgensis intermedia*	Rod.: *Allactaga elater*	Razavi Khorasan (Mashhad)	[26]
		*Paradoxopsyllus grenieri*	Rod.: *Meriones persicus*, *M*. *vinogradovi*	Hamadan (Akanlu)	[21, 26]
		*P*. *microphthalmus*	Rod.: *Meriones persicus*, *Calomyscus bailwardi*	Tehran (Firooz Kooh), Razavi Khorasan (Mashhad)	[21, 26, 28, 44]
		*P*. *tikhomirovae*	Rod.: *Calomyscus bailwardi*, *Meriones persicus*	Isfahan (Ghaleh Tappeh) Kerman, Fars (Shiraz), Razavi Khorasan (Mashhad)	[21, 26]
		*Phaenopsylla tiflovi*	Rod.: *Calomyscus bailwardi*	Isfahan (Ghaleh tappeh), Tehran, West Azarbaijan (Urmia)	[21, 26]
		*P*. *kopetdag*	Rod.: *Calomyscus bailwardi*	Tehran, Mazandaran (Sari), Kerman, Razavi Khorasan (Mashhad)	[26]
	Leptopsyllinae	*Leptopsylla aethiopica aethiopica*	Rod.: *Mus musculus*	Semnan	[50]
		*L*. *putoraki*	Insect: *Crocidura leucodon*	Tehran	[26]
		*L*. *taschenbergi taschenbergi*	Rod.: *Apodemus mystacinus*, *A*. *sylvaticus*, *Rattus rattus*, *Mus musculus*	Hamadan (Razan), Tehran, Gilan (Rasht), Mazandaran (Sari), East Azarbaijan (Tabriz), West Azarbaijan (Urmia), Kermanshah, Fars (Shiraz), Razavi Khorasan (Mashhad), Isfahan	[26, 51]
		*L*. *segnis*	Rod.: *Rattus rattus*, *R*. *norvegicus*, *Mus musculus*	Gilan (Rasht, Bandar Anzali), West Azarbaijan (Urmia), Kermanshah	[21, 26]
		*Peromyscopsylla tikkoinirovae*	Rod.: *Calomyscus bailwardi*	Isfahan (Ghaleh Tappe)	[20]
	Mesopsyllinae	*Caenopsylla laptevi laptevi*	Carn.: *Vulpes vulpes*, Rod.: *Tatera indica*	Isfahan (Shah-Lora, Mahyar), Fars (Shiraz, Kazerun), Markazi (Mahallat), Tehran (Hassan Abad), Kerman	[21, 26, 29, 52]
		*Mesopsylla eucta tuschkan*	Rod.: *Allactaga elater*, *A*. *williamsi*, *Meriones vinogradovi*, *M*. *tristrami M*. *libycus*, *M*. *persicus*, *M*. *libycus*, *Cricetulus migratorius*; Carn.: *Vulpes vulpes* (acc.)	Tehran (Kamal Abad), Hamadan (Agh Bolagh Morshed, Akanlu), Razavi Khorasan (Feiz Abad),	[21, 26]
		*M*. *tuschkan mesa*	Rod.: *Allactaga williamsi*, *A*. *elater*; Carn.: *Vulpes vulpes*	Razavi Khorasan (Mashhad)	[26–27]
		*M*. *tuschkan tuschkan*	Rod.: *Allactaga williamsi*, *A*. *elater*; Carn.: *Vulpes vulpes*	Tehran, Kermanshah	[26–27]
		*M*. *eucta eucta*	Rod.: *Meriones libycus*, *M*. *persicus*	Hamadan (Agh Bolagh Morshed, Akanlu), Razavi Khorasan (Feiz Abad), Tehran (Kamal Abad)	[21, 26]
Pulicidae	Archaeopsyllinae	*Archaeopsylla erinacei erinacei*	Insect.: *Hemiechinus auritus*	Isfahan (Goloon Abad), Hamadan (Agh Bolagh Morshed), Tehran (Youssef Abad),West Azarbaijan (Maku), East Azarbaijan (Maragheh, Marand)	[21, 26, 29, 37]
		*Ctenocephalides canis*	Carn.: *Canis lupus familiaris*, *C*. *lupus pallipes*, *C*. *aureus*, *Vulpes vulpes*, *Meles meles*, *Mustela nivalis*, *Herpestes auropunctatus*, *Hyaena hyaena*; Rod.: *Tatera indica* (acc.); Lag: *Lepus europaeus* (acc.).	Fars (Kazerun), Qazvin, Qom, Hormozgan, Isfahan, Ilam, Kerman, South Khorasan, Kohgilouyeh and Boyerahmad (Margoun, Loudab, Bakhsh-e Markazi), Razavi Khorasan, Lorestan (Khorram-Abad), Khuzestan (Ahvaz, Shoosh, Dezfool, Abadan), Tehran, Zanjan, Mazandaran (Tonekabon, Babolsar), Hamadan (Agh Bolagh Morshed)	[8, 21, 26, 29–30, 32, 53–56]
		*C*. *felis felis*	Carn.: *Canis lupus familiaris*, *Felis catus*, *F*. *silvestris*, *C*. *lupus* spp., *Vulpes vulpes*,*V*. *rueppellii*, *Canis aureus*, *Mustela nivalis*, *Herpestes edwardsii*, *Hyaena hyaena*; Rod.: *Rattus norvegicus* (acc. but not rare); Ungul.: *Ovis aries*, *Capra hircus*	West Azarbaijan, Ilam, Kurdistan, Kohgilouyeh and Boyerahmad (Margoun, Loudab, Bakhsh-e Markazi), Khuzestan (Shoosh, Abadan, Dezfool, Behbahan Ahvaz), Tehran, Golestan (Bandar Torkman), Isfahan, Gilan (Rasht), Fars (Kazerun), Razavi Khorasan (Mashhad)	[8, 21, 26, 32, 53, 57]
		*C*. *orientis*	Carn.: *Canis lupus familiaris*, *C*. *lupus* ssp., *Vulpes vulpes*, *C*. *aureus*, *Felis catus*; Rod.: *Rattus rattus* (acc. but not rare); Ungul.: *Ovis aries*, *Capra hircus*.	Hormozgan (Bandar Jask)	[26]
	Pulicinae	*Echidnophaga gallinacean*	Insect.: *Hemiechinus auritus*, Carn.: *Meles meles* (acc.).	Isfahan (Goloon Abad), Golestan (Bandar Torkman), Tehran (Youssef Abad), Qazvin	[21, 26, 29]
		*E*. *oschanini*	Rod.: *Rhombomys opimus*, *Meriones persicus*, *M*. *libycus*	Isfahan (Yeklengy, Ziar), Golestan (Dash Boroon)	[21, 26, 29]
		*E*. *popovi*	Carn.: *Vulpes vulpes*, *Meles meles*	Hamadan (Agh BolaghMorshed), Tehran (Hesarak), Qazvin	[21, 29]
		*Parapulex chephrenis*	*Acomys* sp.	NS	[27]
		*Pulex irritans*	Prim.: *Homo sapiens*; Carn.: *Canis lupus familiaris*, *C*. *lupus pallipes*, *C*. *aureus*, *Vulpes vulpes*, *Meles meles*, *Herpestes auropunctatus*, *Pantera pardus*, *Hyaena hyaena*; Ungul.: *Ovis aries*, *Capra hircus*, *Bos taurus*; Rod.: *Rattusrattus* (acc.); Insect.: *Hemiechinus auritus*, Artiodact.: *Sus scrofa*; Birds (acc.): *Gallus gallus*, *Corvus corone*	East Azarbaijan, Fars (Shiraz, Kazerun), Ghilan (Assalem), Golestan (Gorgan), Hamadan, Hormozgan, Isfahan (Yeklengi, Ziar, Varzaneh, Shahreza), Kerman, Kermanshah, Khuzestan (Abadan, Shoosh, Khoramshahr, Dezfool, Izeh, Soosangerd), Kohgilouyeh and Boyerahmad, Kurdistan, Lorestan (Khorram-Abad), Markazi, Mazandaran (Marzan Abad, Tonekabon), Persian Golf, Qazvin, Qom, Razavi Khorasan (Mashhad, Ghoochan), Semnan, South Khorasan, Tehran (Hesarak),West Azarbaijan (Miandoab), Zanjan,	[21, 29, 32, 35, 37, 54–55]
	Xenopsyllinae	*Synosternus cleopatrae*	Rod.: *Tatera indica*, *Gerbillus nanus*, *G*. *cheesmani*, Insect.: *Hemiechinus megalotis*	Sistan and Baluchestan (Chabahar, Zabol), Kerman	[26]
		*S*. *pallidus*	Rod.: *Tatera indica*; Insect.: *Hemiechinus auritus*, *H*. *megalotis;* Carn.:*Vulpes vulpes*, *V*. *rueppellii*, *Canis lupus*, *Herpestes auropunctatus*, *Hyaena hyaena*	Khuzestan (Dezfool, Abadan, Shoosh) Bushehr (Borazjan), Tehran (Kamal Abad, Hesarak), Markazi (Mahallat)	[21, 26]
		*Xenopsylla astia*	Rod.: *Rattus norvegicus*, *R*. *rattus*,*Tatera indica*, *Calomyscus bailwardi*, *Meriones crassus*, *M*. *hurrianae*, *Citellus fulvus*, *Nesokia indica*, *Jaculus jaculus*, *Mus musculus*, *Acomys dimidiatus*; Insect.: *Hemiechinus auritus*	Kohgilouyeh and Boyer Ahmad, Kurdistan, Khuzestan (Abadan, Dezfool, Soosangerd, Shoosh), Hormozgan (Bandare Abbas), Kermanshah (Ghasre Shirin), Fars (Kazerun), Razavi Khorasan (Mashhad), Isfahan (Ghale Tappe)	[21, 26, 28, 32, 58–59]
		*X*. *buxtoni*	Rod.: *Rattus norvegicus*, *R*. *rattus*, *Mus musculus*, *Tatera indica*, *Meriones persicus*, *M*. *tristrami*, *M*. *libycus*, *M*. *vinogradovi*, *Microtus arvalis*, *M*. *irani*, *M*. *socialis*, *Nesokia indica*, *Cricetulus migratorius*, *Mesocricetus auratus*, *Allactaga elater*, *A*. *williamsi*; Carn.:*Vulpes vulpes* (acc.), *Meles meles*	Kurdistan, Hormozgan, Lorestan (Khorram-Abad, Weysian, Chaghalvand, Papi, Chegeni, Zagheh), Kermanshah (Sarpole Zahab), Kohgilouyeh and Boyerahmad (Margoun, Loudab, Bakhsh-e Markazi), Tehran (Roudehen Morabad, Ghale Morghi, Talow, Vanak), Qazvin, Markazi (Mahallat), Hamadan (Malayer, Agh Bolagh Morshed, Akanlu), Fars (Kazerun), Qazvin	[21, 26, 32, 34–35, 57–58, 60]
		*X*. *cheopis cheopis*	Rod.: *Rattus norvegicus*, *R*. *rattus*, *Mus musculus*, *Citellus fulvus*, *Cricetulus migratorius*, *Meriones persicus*	Hormozgan (Bandar abbas), Khuzestan (Ahvaz, Shoosh), Gilan (Rasht, Bandar anzali), Golestan (Bandar torkman), Tehran, Isfahan, Razavi Khorasan (Darreh gaz), South Khorasan (Tabas)	[8, 21, 26, 54, 59]
		*X*. *conformis conformis*	Rod.: *Allactaga elator*, *Tatera indica*, *Rhombomys opimus*, *Jaculus jaculus*, *J*. *blanfordi*, *Gerbillus nanus*, *Meriones tristrami*, *M*. *crassus*, *M*. *persicus*, *M*. *vinogradovi*, *M*. *tristrami*, *M*. *meridianus*, *M*. *libycus*, *Citellus fulvus*, *Cricetulus migratorius*, *Nesokia indica*, *Calomyscus bailwardi*; Insect.: *Hemiechinus megalotis*	Sistan and Baluchestan (Daman, Qasre Qand, Bampour), Kurdistan, Isfahan (Yeklengy, Ghaleh tappeh), Tehran (Hassan Abad, Kan, Najm Abad, Kamal Abad, Hesarak, Seid Abad), Razavi Khorasan (Mashhad, Mosen Abad, Feiz Abad, Darreh Gaz, Sange Atesh), Khuzastan (Shoosh), South Khorasan (Tabas), Kermanshah (Shahmar), Golestan (Dash Boroon), Qazvin, Markazi (Aziz Abad)	[21, 26, 28–29, 32, 58]
		*X*. *conformis mycerini*	Rod.: *Allactaga elator*	Razavi Khorasan (Mashhad)	[28, 44]
		*X*. *gerbilli gerbilli*	Rod.: *Rhombomys opimus*	Razavi Khorasan (Darreh Gaz, Lotf Abad)	[26]
		*X*. *hussaini*	Rod.: *Nesokia indica*, *Tatera indica*, *Gerbillus nanus*, *G*. *cheesmani*, *Meriones persicus*, Insect.: *Hemiechinus megalotis*	Fars (Shiraz), Kerman	[26]
		*X*. *nubica*	Rod.: *Allactaga elator*, *Jaculus jaculus*, *J*. *blanfordi*	Sistan and Baluchestan (Daman, Qasre Qand, Bampour), Isfahan (Harrand)	[26, 28–29, 58]
		*X*. *nuttalli*	Rod.: *Rhombomys opimus*, *Gerbillus nanus*,*G*. *cheesmani*, *Meriones persicus*, *M*. *crassus*, *M*. *meridianus*, *Nesokia indica*, *Hystrix indica*, *Mus musculus*, *Calomyscus bailwardi*, *Tatera indica*, *Ellobius fuscocapillus;* Insect.: *Hemiechinus auritus;* Carn.: *Vulpes vulpes;* Ungul.: *Ovis aries*, *Bos taurus*, *Capra hircus*; Lag.: *Lepus capensis*, *Ochotona rufescens*, Chi.: *Pipistrellus pipistrellus* (acc.)	Kohgilouyeh and Boyerahmad (Bakhsh-e Markazi, Margoun, Loudab), Isfahan (Shahreza), Golestan (Dash Boroon), Razavi Khorasan (Sabzehevar)	[21, 26, 29, 32, 61]
		*X*. *persica*	Rod.: *Meriones persicus*, *Rhombomys opimus*	Razavi Khorasan (Asi Bolagh Ghoochan)	[5, 26]
Vermipsyllidae	NS	*Chaetopsylla globiceps*	Carn.: *Vulpes vulpes*	Isfahan (Shah-Lora, Khansar, Shahreza) Mashhad, Fars (Shiraz)	[21, 26, 29]
		*C*. *hyaenae*	Carn.: *Hyaena hyaena*	Tehran	[21, 26]
		*C*. *korobkovi*	Carn.: *Vulpes vulpes*	Hamadan (Agh Bolagh Morshed, Akanlu) Isfahan (Shahreza, Shah-Lora), Fars (Shiraz)	[21, 26, 29]
		*C*. *trichosa avicenni* (= *C*. *trichosa)*	Carn.: *Meles meles*	Kurdistan	[21, 44]

**Abbreviations**: Carn.: Carnivora, Rod.: Rodentia, Lag.: Lagomorpha, Insect.: Insectivora, Ungul.: Ungulate, Prim.: Primates, Chi.: Chiroptera, Artiodact.: Artiodactyla, acc.: accidental host. NS: Not Stated. * In the old classification of Iran provinces which implied by Farhang-Aazad (1972b)[62], the main cities were regarded as flea collection locality [26].

**Table 2:** Comparison of taxonomic data on Siphonaptera and their host orders represented in Iran with the counterpart ones in the world [5, 63].

Family	Distribution (region)	Subfamily		Genera		Species / Subspecies		Major host	
		World	Iran	World	Iran	World	Iran	World	Iran
Ceratophyllidae	Cosmopolitan but predominantly Holarctic	2	1	44	6	403	33	Primarily rodents, occasionally viverrids, mustelids, birds, and a single species on an insectivore (Siberian mole)	Rodentia (71.05%) Birds (10.53%), Insectivora (7.89%), Carnivora (5.26%) and Lagomorpha (5.26%)
Coptopsyllidae	Palearctic	0	0	1	1	19	9	Rodents (gerbils and their allies)	Rodentia (90.91%) and Carnivora (9.09%)
Ctenophthalmidae	Primarily Holarctic, and Afrotropical some in southern hemisphere	9	6	42	7	548	20	Rodents, occasionally pikas, insectivores (shrews and moles), marsupials, and a single species on mustelids	Rodentia (75.86%), Insectivora (17.24%) and Carnivora (6.9%)
Ischnopsyllidae	Cosmopolitan	2	1	20	3	122	6	Chiroptera	Chiroptera (100%)
Leptopsyllidae	Palearctic, Nearctic, Oriental, a few species in Australian or Ethiopian regions	*2	*3	29	10	230	24	Rodents, lagomorphs (hares, rabbits, pikas), insectivores, and rarely elephant shrews and foxes	Rodentia (80.95%), Insectivora (9.52%), Lagomorpha (4.76%) Carnivora (4.76%)
Pulicidae (includes erroneously tungid flea)	Cosmopolitan	6	3	27	7	182	21	Very broad host range, including carnivores, ungulates, bats, edentales (armadillos), and occasionally birds (*Cariama* spp).	Rodentia (52.63%), Ungulates (5.26%), Carnivora (24.56%), Artiodactyla (1.75%), Insectivora (3.51%), Birds (3.51%), Chiroptera (1.75%), Primates (1.75%) and Lagomorpha (5.26%)
Vermipsyllidae	Holarctic	0	0	3	1	39	4	Carnivores and ungulates	Carnivora (100%)
Total		21	14	166	35	1543	117		

* Altough Leptopsyllidae further classified as subfamilies Amphipsyllinae and Leptopsyllinae and the tribes Amphipsyllini and Leptopsyllini [64] but Klein (1963) was regarded Mesopsyllinae as third subfamily in the Leptopsyllidae of Iran [21].

The Ceratophyllidae, the more represented flea family, consisted of 33 species belonging to 6 genera, comprising *Callopsylla*, *Ceratophyllus*, *Citellophilus*, *Myoxopsylla*, *Nosopsyllus* and *Paraceras*.

The Leptopsyllidae, bird and rodent fleas, consisted of 24 species consisting of 10 genera including *Amphipsylla*, *Caenopsylla*, *Ctenophyllus*, *Frontopsylla*, *Leptopsylla*, *Mesopsylla*, *Ophthalmopsylla*, *Paradoxopsyllus*, *Peromyscopsylla* and *Phaenopsylla*.

The Ctenophthalmidae consisted of 20 species belonging to 7 genera comprising *Ctenophthalmus*, *Doratopsylla*, *Neopsylla*, *Palaeopsylla*, *Rhadinopsylla*, *Stenoponia* and *Wagnerina*.

The Pulicidae, a cosmopolitan family of the most notorious plague vectors (genus *Xenopsylla*), included 21 species distributed in 7 genera comprising *Archaeopsylla*, *Ctenocephalides*, *Echidnophaga*, *Pulex*, *Synosternus*, *Parapulex*, and *Xenopsylla*.

The Coptopsyllidae was limited to 9 species in the genus *Coptopsylla*.

In the above-mentioned five families, the most commonly reported fleas belong to the genera *Nosopsyllus* (Ceratophyllinae), *Xenopsylla* (Xenopsyllinae), *Ctenophthalmus* (Ctenophthalminae) *Coptopsylla* (Coptopsyllidae) *Amphipsylla* (Amphipsyllinae), *Leptopsylla* (Leptopsyllinae), and *Mesopsylla* (Mesopsyllinae). Detailed information is presented in Table 1.

### Host diversity and associated flea fauna

The hosts associated with Iran fleas ranged from the small mammals (Rodentia, Chiroptera, Lagomorpha, Insectivora) to the large mammals (Ungulata, Carnivora, Primates, Artiodactyla) and birds as well. On the whole, 166 vertebrate host species were reported infested by fleas in Iran in the literature including Pulicidae (n = 56), Ceratophyllidae (n = 38), Ctenophthalmidae (n = 29), Leptopsyllidae (n = 22), Coptopsyllidae (n = 11), Ischnopsyllidae (n = 7) and Vermipsyllidae (n = 3). By filtering the compiled data, we recognized 77 vertebrate host species among all seven flea families.

Eight potential mammals were hosted by ≤7 flea (sub-) family respectively; these were: *Calomyscus bailwardi* (7), *Meles meles* (7), *Mus musculus* (7), *Meriones vinogradovi* (8), *Vulpes vulpes* (8), *Cricetulus migratorius* (9), *Meriones libycus* (9) and *Meriones persicus* (11). Actually flea (sub-) families can infest ≥10 vertebrate hosts were Xenopsyllinae (n = 43), Ceratophyllinae (n = 37), Archaeopsyllinae (n = 20), Ctenophthalminae (n = 20), Pulicinae (n = 19), Amphipsyllinae (n = 17), Stenoponiinae (n = 12) and Coptopsyllidae (n = 11). Detailed information is presented in Table 3.

**Table 3:** Common mammal hosts in Iran and their flea diversity at the family and subfamily levels.

Host	Typical habitat	(sub-) family															Total flea (sub-) families per host
		Ce	Co	An	Ct	Do	Ne	Rh	St	Am	Le	Me	Ar	Pu	Xe	Ve	
*Acomys dimidiatus*	semi-arid or dry habitats	-	-	-	-	-	-	-	-	-	-	-	-	-	1	-	1
*Allactaga elater*	deserts and semideserts	-	-	-	-	-	-	-	-	1	-	1	-	-	1	-	3
*Allactaga williamsi*	steppe regions with sparse vegetation	1	-	-	1	-	-	-	-	1	-	1	-	-	1	-	5
*Apodemus mystacinus*	forest with rocky areas	-	-	-	1	1	-	-	-	1	1	-	-	-	-	-	4
*Apodemus sylvaticus*	wide variety of semi-natural habitats	-	-	-	1	-	-	-	-	-	1	-	-	-	-	-	2
*Bos Taurus*	rangelands	-	-	-	-	-	-	-	-	-	-	-	-	1	1	-	2
*Calomyscus bailwardi*	barren, dry and rocky mountainsides	1	1	1	-	-	-	-	1	1	1	-	-	-	1	-	7
*Canis aureus*	dry habitats	-	-	-	-	-	-	-	-	-	-	-	1	1	-	-	2
*Canis lupus* spp.	arid desert regions to dense scrub forests	-	-	-	-	-	-	-	-	-	-	-	1	-	1	-	2
*Canis lupus familiaris*	northern habitats with sufficient prey	-	-	-	-	-	-	-	-	-	-	-	1	1	-	-	2
*Canis lupus pallipes*	northern habitats with sufficient prey	-	-	-	-	-	-	-	-	-	-	-	1	1	-	-	2
*Capra hircus*	rangelands	-	-	-	-	-	-	-	-	-	-	-	1	1	1	-	3
*Citellus fulvus*	deserts and semi-deserts	1	-	-	-	-	1	-	1	-	-	-	-	-	1	-	4
*Cricetulus migratorius*	arid or semi-arid regions	1	1	1	1	-	1	1	-	1	-	1	-	-	1	-	9
*Crocidura leucodon*	moist mountainous regions	1	-	-	1	-	-	-	-	-	1	-	-	-	-	-	3
*Crocidura suaveolens*	arid areas with moist vegetation	1	-	-	1	1	-	-	-	-	-	-	-	-	-	-	3
*Dryomys nitedula*	broad variety of woodlands	1	-	-	-	-	-	-	-	-	-	-	-	-	-	-	1
*Ellobius fuscocapillus*	open steppes habitat with loose soil	1	-	-	-	-	-	-	-	-	-	-	-	-	1	-	2
*Felis catus*	cosmopolitan domestic species	-	-	-	-	-	-	-	-	-	-	-	1	-	-	-	1
*Felis silvestris*	areas with rocks and tall trees	-	-	-	-	-	-	-	-	-	-	-	1	-	-	-	1
*Gerbillus cheesmani*	sandy soils and mud flats	1	-	-	-	-	-	-	-	-	-	-	-	-	1	-	2
*Gerbillus dasyurus*	desert, semi-desert, and rocky habitats	-	-	-	-	-	-	-	1	-	-	-	-	-	-	-	1
*Gerbillus nanus*	desert, semi-desert, arable land and gardens	1	1	-	-	-	-	-	1	-	-	-	-	-	1	-	4
*Hemiechinus auritus*	dry steppes, semi-deserts and deserts	1	-	-	-	-	-	-	-	1	-	-	1	1	1	-	5
*Hemiechinus megalotis*	mountainous areas	-	-	-	-	-	-	-	-	-	-	-	-	-	1	-	1
*Herpestes auropunctatus*	scrublands and dry forest	-	-	-	-	-	-	-	-	-	-	-	1	1	1	-	3
*Herpestes edwardsii*	thickets, cultivated fields or broken, bushy vegetation	-	-	-	-	-	-	-	-	-	-	-	1	-	-	-	1
*Homo sapiens*	passim	-	-	-	-	-	-	-	-	-	-	-	-	1	-	-	1
*Hyaena hyaena*	arid to semi-arid environments	-	-	-	-	-	-	-	-	-	-	-	1	1	1	1	4
*Hystrix indica*	broad range of habitats	-	-	-	-	-	-	-	-	-	-	-	-	-	1	-	1
*Jaculus blanfordi*	desert with clay soil	1	-	-	-	-	-	-	-	-	-	-	-	-	1	-	2
*Jaculus Jaculus*	sandy or rocky deserts	1	-	-	-	-	-	-	-	-	-	-	-	-	1	-	2
*Lepus capensis*	Shrubs to open habitats	1	-	-	-	-	-	-	-	-	-	-	-	-	1	-	2
*Lepus europaeus*	open fields and pastures	-	-	-	-	-	-	-	-	-	-	-	1	-	-	-	1
*Meles meles*	woodlands	1	1	-	-	-	-	-	1	-	-	-	1	1	1	1	7
*Meriones crassus*	dry habitats in sandy deserts	1	1	-	-	-	-	-	1	-	-	-	-	-	1	-	4
*Meriones hurrianae*	sandy plains with higher density of bushes	-	-	-	-	-	-	-	-	-	-	-	-	-	1	-	1
*Meriones libycus*	arid or semi-arid regions	1	1	-	1	-	-	1	1	1	-	1	-	1	1	-	9
*Meriones meridianus*	sand deserts	1	1	-	-	-	-	-	-	-	-	-	-	-	1	-	3
*Meriones persicus*	arid, rocky, mountainous region	1	1	1	1	-	1	1	1	1	-	1	-	1	1	-	11
*Meriones tristrami*	steppe and semi-desert habitats	1	-	-	1	-	-	1	1	-	-	1	-	-	1	-	6
*Meriones vinogradovi*	semi desert, bare mountains and wastelands	1	1	-	1	-	-	1	1	1	-	1	-	-	1	-	8
*Mesocricetus auratus*	arable fields with annual crops	1	-	1	1	-	-	1	-	1	-	-	-	-	1	-	6
*Microtus arvalis*	moist meadows, moist and forest steppe, agricultural areas	1	-	-	1	-	-	-	-	1	-	-	-	-	1	-	4
*Microtus irani*	mountainous ranges	1	-	-	1	-	-	1	-	1	-	-	-	-	1	-	5
*Microtus nivalis*	mountainous ranges	1	-	-	-	-	-	-	-	-	-	-	-	-	-	-	1
*Microtus socialis*	steppe habitats	1	-	-	1	-	-	1	-	1		-	-	-	1	-	5
*Mus musculus*	cosmopolitan domestic species	1	-	1	1	-	-	1	-	1	1	-	-	-	1	-	7
*Mustela nivalis*	wide range of habitats	-	-	-	-	-	-	-	-	-	-	-	1	-	-	-	1
*Nesokia indica*	dry deciduous forests, scrublands, grasslands, arable land, pastures, plantations	1	-	1	1	-	-	-	-	-	-	-	-	-	1	-	4
*Ochotona rufescens*	mountainous regions	1	-	-	-	-	-	-	-	1	-	-	-	-	1	-	3
*Ovis aries*	cosmopolitan domestic species	-	-	-	-	-	-	-	-	-	-	-	1	1	1	-	3
*Pantera pardus*	wide range of habitats	-	-	-	-	-	-	-	-	-	-	-	-	1	-	-	1
*Pipistrellus pipistrellus*	wide range of habitats (Gorgan city, *Golestan* Province)	-	-	-	-	-	-	-	-	-	-	-	-	-	1	-	1
*Pitymys majori*	mixed forests	-	-	-	1	-	-	-	-	-	-	-	-	-	-	-	1
*Rattus norvegicus*	lowland and coastal regions	1	-	-	-	-	-	-	-	1	1	-	1	-	1	-	5
*Rattus rattus*	natural and semi-natural habitats	1	-	-	-	-	-	-	-	-	1	—	1	1	1	-	5
*Rhombomys opimus*	desert to semi-desert habitats	1	1	-	-	-	-	-	1	-	-	-	-	1	1	-	5
*Sorex minutes*	wide variety of habitats	-	-	-	-	1	-	-	-	-	-	-	-	-	-	-	1
*Sus scrofa*	a wide variety of temperate and tropical habitats	-	-	-	-	-	-	-	-	-	-	-	-	1	-	-	1
*Talpa caeca*	deciduous woodland, meadows and pastures in hilly or mountainous areas	-	-	-	1	-	-	-	-	-	-	-	-	-	-	-	1
*Talpa europaea*	deep soils	-	-	-	1	-	-	-	-	-	-	-	-	-	-	-	1
*Tatera indica*	arid habitats	1	1	-	-	-	-	-	1	-	-	1	1	-	1	-	6
*Vulpes rueppellii*	sand and stony deserts	-	-	-	-	-	-	-	-	-	-		1	-	1	-	2
*Vulpes vulpes*	tundra, desert and forest, as well as in city centres	1	-	-	1	-	-	-	-	1	-	1	1	1	1	1	8
Total host/flea (sub-) families		37	11	6	20	3	3	9	12	17	7	9	20	19	43	3	215 219

**Ce** Ceratophyllinae, **Co** Coptopsyllidae, **An** Anomiopsyllinae, **Ct** Ctenophthalminae, **Do** Doratopsyllinae, **Ne** Neopsyllinae, **Rh** Rhadinopsyllinae, **St** Stenoponiinae, **Am** Amphipsyllinae, **Le** Leptopsyllinae, **Me** Mesopsyllinae, **Ar** Archaeopsyllinae, **Pu** Pulicinae, **Xe** Xenopsyllinae and **Ve** Vermipsyllidae

At least 23, 6, 5, 5 and 1 host species are exclusively infested by Pulicidae, Ischnopsyllidae, Ceratophyllidae, Ctenophthalmidae and Leptopsyllidae respectively. However restricted host species was not found in the Coptopsyllidae and Vermipsyllidae (Table 4).

**Table 4:** Shared and exclusive vertebrate species associated with seven flea families of Pulicidae, Ceratophyllidae, Ctenophthalmidae, Leptospyllidae, Coptopsyllidae, Ischnopsyllidae and Vermipsyllidae in Iran.

Flea family(s)	No of host(s)	Vertebrate host(s)
Ceratophyllidae, Coptopsyllidae, Ctenophthalmidae, Leptopsyllidae and Pulicidae	6	*Calomyscus bailwardi*, *Cricetulus migratorius*, *Meriones libycus*, *Meriones persicus*, *Meriones vinogradovi* and *Tatera indica*
Ceratophyllidae, Coptopsyllidae, Ctenophthalmidae, Pulicidae and Vermipsyllidae	1	*Meles meles*
Ceratophyllidae, Ctenophthalmidae, Leptopsyllidae, Pulicidae and Vermipsyllidae	1	*Vulpes vulpes*
Ceratophyllidae, Coptopsyllidae, Ctenophthalmidae and Pulicidae	3	*Gerbillus nanus*, *Meriones crassus* and *Rhombomys opimus*
Ceratophyllidae, Ctenophthalmidae, Leptopsyllidae and Pulicidae	7	*Allactaga williamsi*, *Meriones tristrami*, *Mesocricetus auratus*, *Microtus arvalis*, *Microtus irani*, *Microtus socialis* and *Mus musculus*
Ceratophyllidae, Coptopsyllidae and Pulicidae	1	*Meriones meridianus*
Ceratophyllidae, Ctenophthalmidae and Leptopsyllidae	1	*Crocidura leucodon*
Ceratophyllidae, Ctenophthalmidae and Pulicidae	2	*Citellus fulvus*, *Nesokia indica*
Ceratophyllidae, Leptopsyllidae and Pulicidae	4	*Hemiechinus auritus*, *Ochotona rufescens*, *Rattus norvegicus* and *Rattus rattus*
Ceratophyllidae and Ctenophthalmidae	1	*Crocidura russula*
Ceratophyllidae and Pulicidae	6	*Ellobius fuscocapillus*, *Gallus gallus*, *Gerbillus cheesmani*, *Jaculus blanfordi*, *Jaculus jaculus* and *Lepus capensis*
Ctenophthalmidae and Leptopsyllidae	2	*Apodemus mystacinus* and *Apodemus sylvaticus*
Ischnopsyllidae and Pulicidae	1	*Pipistrellus pipistrellus*
Pulicidae and Vermipsyllidae	1	*Hyaena hyaena*
Ceratophyllidae	5	*Dryomys nitedula*, *Galerida cristata*, *Microtus nivalis*, *Motacilla alba* and *Passer domesticus*
Ctenophthalmidae	5	*Gerbillus dasyurus*, *Pitymys majori*, *Sorex minutes*, *Talpa caeca* and *Talpa europaea*
Ischnopsyllidae	6	*Aselia tridens*, *Myotis blythi*, *Nyctalus noctula*, *Pipistrellus kuhlii*, *Plecotus macrobullaris* and *Rhinolophus blasii*
Leptopsyllidae	1	*Allactaga elater*
Pulicidae	23	*Acomys dimidiatus*, *Allactaga elator*, *Bos taurus*, *Canis aureus*, *Canis lupus familiaris*, *Canis lupus pallipes*, *Canis lupus* spp., *Capra hircus*, *Corvus corone*, *Felis catus*, *Felis silvestris*, *Hemiechinus megalotis*, *Herpestes auropunctatus*, *Herpestes edwardsi*, *Homo sapiens*, *Hystrix indica*, *Lepus europaeus*, *Meriones hurrianae*, *Mustela nivalis*, *Ovis aries*, *Pantera pardus*, *Sus scrofa* and *Vulpes ruppelli*

A total of 53 vertebrate species were reported infested by six subfamilies of Ctenophthalmidae including Ctenophthalminae (n = 20), Stenoponiinae (n = 12), Rhadinopsyllinae (n = 9), Anomiopsyllinae (n = 6), Doratopsyllinae (n = 3) and Neopsyllinae (n = 3). By filtering the compiled data, 29 vertebrate host species were distinguished among all six subfamilies. Correspondingly 8, 6 and 1 host species are exclusively included in the Ctenophthalminae, Stenoponiinae and Doratopsyllinae. However there were not found any restricted vertebrate host species in the Anomiopsyllinae, Neopsyllinae and Rhadinopsyllinae (Table 5).

**Table 5:** Shared and exclusive vertebrate species associated with six subfamilies of Ctenophthalmidae in Iran.

Flea sub family(s)	No of host (s)	Vertebrate host(s)
Anomiopsyllinae, Ctenophthalminae, Neopsyllinae, Rhadinopsyllinae and Stenoponiinae	1	*Meriones persicus*
Anomiopsyllinae, Ctenophthalminae, Neopsyllinae and Rhadinopsyllinae	1	*Cricetulus migratorius*
Anomiopsyllinae, Ctenophthalminae and Rhadinopsyllinae	2	*Mesocricetus auratus* and *Mus musculus*
Ctenophthalminae, Rhadinopsyllinae and Stenoponiinae	3	*Meriones libycus*, *Meriones tristrami* and *Meriones vinogradovi*
Anomiopsyllinae and Ctenophthalminae	1	*Nesokia indica*
Anomiopsyllinae and Stenoponiinae	1	*Calomyscus bailwardi*
Ctenophthalminae and Doratopsyllinae	2	*Apodemus mystacinus* and *Crocidura russula*
Ctenophthalminae and Rhadinopsyllinae	2	*Microtus irani* and *Microtus socialis*
Neopsyllinae and Stenoponiinae	1	*Citellus fulvus*
Ctenophthalminae	8	*Allactaga williamsi*, *Apodemus sylvaticus*, *Crocidura leucodon*, *Microtus arvalis*, *Pitymys majori*, *Talpa caeca*, *Talpa europaea* and *Vulpes vulpes*
Doratopsyllinae	1	*Sorex minutes*
Stenoponiinae	6	*Gerbillus dasyurus*, *Gerbillus nanus*, *Meles meles*, *Meriones crassus*, *Rhombomys opimus* and *Tatera indica*

A total of 33 vertebrate species were reported infested by three subfamilies of Leptopsyllidae including Amphipsyllinae (n = 17), Mesopsyllinae (n = 9) and Leptopsyllinae (n = 7). By filtering the compiled data, 22 vertebrate host species were distinguished among three subfamilies. Investigation on the flea-host associations in subfamilies of the Leptopsyllidae showed that there were no common host species shared by the three subfamilies. However 6, 3 and 2 host species are exclusively included in the Amphipsyllinae, Leptopsyllinae and Mesopsyllinae respectively (Table 6).

**Table 6:** Shared and exclusive vertebrate species associated with three subfamilies of Ctenophthalmidae in Iran.

Flea sub family(s)	No of host (s)	Vertebrate host(s)
Amphipsyllinae and Leptopsyllinae	4	*Apodemus mystacinus*, *Calomyscus bailwardi*, *Mus musculus* and *Rattus norvegicus*
Amphipsyllinae and Mesopsyllinae	7	*Allactaga elater*, *Allactaga williamsi*, *Cricetulus migratorius*, *Meriones libycus*, *Meriones persicus*, *Meriones vinogradovi* and *Vulpes vulpes*
Amphipsyllinae	6	*Hemiechinus auritus*, *Mesocricetus auratus*, *Microtus arvalis*, *Microtus irani*, *Microtus socialis* and *Ochotona rufescens*
Leptopsyllinae	3	*Apodemus sylvaticus*, *Crocidura leucodon* and *Rattus rattus*
Mesopsyllinae	2	*Meriones tristrami* and *Tatera indica*

A total of 83 vertebrate species were reported infested by three subfamilies of Pulicidae including Xenopsyllinae (n = 43), Pulicinae (n = 20) and Archaeopsyllinae (n = 20). By filtering the compiled data, 56 vertebrate host species were distinguished among three subfamilies. Exploration of flea-host associations in Pulicidae pointed out that there are eight common hosts including *Capra hircus* (Linnaeus, 1758), *Hemiechinus auritus* (Gmelin, 1770), *Herpestes auropunctatus* (Hodgson, 1836), *Hyaena hyaena* (Linnaeus, 1758), *Meles meles* (Linnaeus, 1758), *Ovis aries* (Linnaeus, 1758), *Rattus rattus* (Linnaeus, 1758) and *Vulpes vulpes* (Linnaeus, 1758) among three subfamilies. Although a number of 27, 5 and 5 host species are exclusively included in the Xenopsyllinae, Pulicinae and Archaeopsyllinae respectively (Table 7).

**Table 7:** Shared and exclusive vertebrate species associated with three subfamilies of Pulicidae in Iran.

Flea sub family(s)	No of host (s)	Vertebrate host(s)
Archaeopsyllinae, Pulicinae and Xenopsyllinae	8	*Capra hircus*, *Hemiechinus auritus*, *Herpestes auropunctatus*, *Hyaena hyaena*, *Meles meles*, *Ovis aries*, *Rattus rattus* and *Vulpes vulpes*
Archaeopsyllinae and Pulicinae	3	*Canis aureus*, *Canis lupus familiaris* and *Canis lupus pallipes*
Archaeopsyllinae and Xenopsyllinae	4	*Canis lupus* spp., *Rattus norvegicus*, *Tatera indica* and *Vulpes ruppelli*
Pulicinae and Xenopsyllinae	4	*Bos Taurus*, *Meriones libycus*, *Meriones persicus* and *Rhombomys opimus*
Archaeopsyllinae	5	*Felis catus*, *Felis silvestris*, *Herpestes edwardsi*, *Lepus europaeus* and *Mustela nivalis*
Pulicinae	5	*Corvus corone*, *Gallus gallus*, *Homo sapiens*, *Pantera pardus* and *Sus scrofa*
Xenopsyllinae	27	*Gerbillus cheesmani*, *Hystrix indica*, *Allactaga williamsi*, *Calomyscus bailwardi*, *Microtus irani*, *Cricetulus migratorius*, *Microtus arvalis*, *Jaculus jaculus*, *Ochotona rufescens*, *Gerbillus nanus*, *Pipistrellus pipistrellus*, *Nesokia indica*, *Meriones vinogradovi*, *Mesocricetus auratus*, *Mus musculus*, *Meriones crassus*, *Allactaga elater*, *Ellobius fuscocapillus*, *Acomys dimidiatus*, *Hemiechinus megalotis*, *Meriones tristrami*, *Citellus fulvus*, *Meriones hurrianae*, *Microtus socialis*, *Jaculus blanfordi*, *Lepus capensis*, and *Meriones meridianus*

## Discussion

The literature inventory of the fleas of Iran showed that there are seven Siphonaptera families in this country namely Ceratophyllidae, Leptopsyllidae, Pulicidae, Ctenophthalmidae, Coptopsyllidae, Ischnopsyllidae and Vermipsyllidae. These flea families are distributed in all parts of the country where sampling occurred and where data were available. According to the literature reviewed, the distribution range of those families extends in Hamadan and Kurdistan (West Iran) provinces rather than in Ardabil (northwest), Northern Khorasan (northeast), Bushehr (south), Mazandaran, Golestan and Gilan provinces (north). This fact is partly due to a collection bias in plague foci during the sixties (1963–1975 Baltazard, Klein, Farhang-Azad and Mollaret)[65–70]. The distribution maps of the studied fleas showed that further sampling, especially from provinces with poor faunistical studies, is necessary, especially in a context of vector-borne disease epidemiology where known mammalian hosts of pathogenic agents are also present.

Most fleas of medical or veterinary importance belong to the Ceratophyllidae, Leptopsyllidae, Pulicidae, Ctenophthalmidae and Vermipsyllidae families [12]. Pulicidae, a family including most cosmopolitan flea species of medical importance and in particular the *Xenopsylla* genus, was by far the most reported family in Iran [8, 29–30, 32, 35, 53–55, 57–60]. Analysis of common mammal hosts and their flea diversity revealed that *M*. *persicus* was infested by 11 flea subfamilies and Xenopsyllinae were hosted by at least 43 mammal species.

The Persian Jird, *M*. *persicus*, is distributed from Eastern Anatolia to Afghanistan and western Pakistan. Iran is the most extensive geographical region in the distribution range of the Persian Jird; indeed five of the six subspecies are found in the country [71].

At the first, the research team of Baltazard (1952) and then Golvan & Rioux (1963) and Poland and Dennis (1999) offered initial illustrations of the role of resistant or silent enzootic reservoirs in the maintenance of *Y*. *pestis* and human plague outbreaks in the Kurdistan focus. They showed that *M*. *vinogradovi* and *M*. *tristrami* were extremely sensitive to *Y*. *pestis* while *M*. *libycus* and *M*. *persicus* were highly resistant. *Tatera indica* has also been associated with transmission of *Y*. *pestis* in the country. Flea densities were reported to be high on *M*. *persicus* [23, 72–73]. In that era flea species including *Pulex irritans*, *Xenopsylla cheopis*, *X*. *astia*, *X*. *buxtoni*, *X*. *conformis*, *Nosopsyllus fasciatus N*. *iranus iranus*, and *Stenoponia tripectinata* were listed as favorite candidate *Y*. *pestis* vectors within and among vertebrates including man [74–79].

In 1980, Karimi et al. surveyed the Sarab focus in East Azarbaijan province where fourteen samples of *Y*. *pestis* were isolated from *M*. *persicus*, *M*. *vinogradovi*, and *Mesocricetus auratus* and from their fleas; *Xenopsylla conformis* and *Nosopsylla iranus iranus* [80]. The *Y*. *pestis* strains isolated from the *M*. *persicus* in the Trans-Arax focus in Armenia were characterized by higher virulence than those that are isolated from voles in the Transcaucasus Mountainous focus[81].

In a recent serological survey carried out by Esmaeili et al., in Western Iran antibodies against *Y*. *pestis* F1 capsular antigen were detected in a *M*. *persicus* [22]. Whether *Y*. *pestis* strains lacking the F1 antigen naturally occur in Iran is not known but could lead to an underestimation of the current seroprevalence.

*Meriones* species notably *M*. *persicus* were reported to be main reservoir host for pathogens rather than bacterium *Y*. *pestis*. In the parasitological studies sandfly-borne *Leishmania* spp. including *L*. *major* [82], *L*. *infantum* [83] and *L*. *donovani* [84] were isolated from *M*. *persicus* specimens. *Meriones* species rather than *M*. *persicus* (*M*. *libycus* and *M*. *hurrianae*) have been reported as the major reservoir host of zoonotic cutaneous leishmaniasis in several endemic areas of Iran [85–89]. The endoparasites ranging from Acanthocephala to Cestoda and Nematoda were identified in *M*. *persicus* as well [90]. These findings place the Persian jird and the Xenopsyllinae as the major vertebrate and vector hosts of flea-borne diseases in Iran including *Y*. *pestis*, the etiological agent of plague.

Indeed, *Xenopsylla* spp. were collected from 18 provinces with a wide array of climatic conditions ranging from cold mountainous areas to warm and dry sandy plains and deserts (Table 1).

Most species of the Pulicidae family are notorious vectors of disease agents causing plague, murine typhus, and tularemia but also transmit helminths. Several species of the *Xenopsylla* genus play an important role in the transmission of *Y*. *pestis*, the etiological agent of plague, from rodents to human [91]; the most classical and significant vector being *X*. *cheopis* [92].

Indeed, *X*. *cheopis* accounts for 80% of the fleas collected off rodent hosts in the natural endemic plague foci of Iran [93]. *X*. *cheopis* is also the vector of various human pathogenic *Bartonella* species [6, 94]. The cat scratch disease, caused by *B*. *henselae*, has been considered as an emerging zoonotic bacterial pathogen in veterinary and human medicine. Cats are the basic source of the bacteria. Bacteria are transferred from cat to cat by the flea *Ctenocephalides felis*, another cosmopolitan flea, which have been reported in the Iranian cat population [95]. Murine typhus or endemic typhus caused primarily by *Rickettsia typhi*is another rodent-borne disease that is transmitted to humans by the flea *X*. *cheopis* [96]. *Pulex irritans* and *Nosopsyllus fasciatus* are secondary vectors of murine typhus *Rickettsia* [97] that is endemic through coastal regions of the Caspian Sea and the Persian Gulf [98].

*Rickettsia felis* is the cause of another flea-borne “spotted fever group” rickettsiosis. *R*. *felis* is transmitted by the bite or faeces of several flea species, and transovarially in *Ctenocephalides felis felis* (and the African subspecies *C*. *f*. *strongylus*) but also in *C*. *orientis* present in Iran, so that they are considered as vectors and reservoir hosts of this pathogen [99].

*Ctenocephalides felis*, *C*. *canis*- that have been collected from the studied areas extensively (Table 1)—and *P*. *irritans* are the intermediate hosts of flatworms such as *Dipylidium caninum*, or nematodes as the filaria *Acanthocheilonema reconditum*. Hence dog, cat and rarely human infection occurs following ingestion of infected fleas [100–101]. Typically, a human is bitten more often by a cat flea (*C*. *felis*) than a dog flea (*C*. *canis*) which is very or even monospecific. Cosmopolitan fleas as helminths vector have less medical than veterinary importance, since the helminth species they transmit rarely infest humans and are virtually harmless.

*Nosopsyllus fasciatus*, a Ceratophyllidae and *Coptopsylla lamellifer*, a Coptopsyllidae, were collected in 14 different regions of Iran. They play a role in enzootic plague cycles, that is in circulating the plague bacterium *Y*. *pestis* between rodents but since they do not readily bite humans in a natural setting, are only accidental vectors of *Y*. *pestis* to humans exposed [38, 41, 102–103].

Fleas are also considered vectors of *F*. *tularensis* the etiological agent of tularemia [104]. Vulnerable animals such as hares and rodents frequently die in large numbers during epizootics. Human infections take place through several routes, including insect bites and direct contact to an infected animal. It can affect the skin, eyes, lymph nodes, lungs and, less often, other internal organs. According to recent studies (which have shown the presence of this disease in western and eastern regions of Iran) and the previous studies (which have shown the presence of this disease in the east and north-west of the country [105]), the possibility of transmission of this agent by fleas should be considered in all parts of the country [106].

Most leptopsyllids parasitize rodents and a few birds. Species of *Frontopsylla*, *Leptopsylla*, *Mesopsylla*, *Ophthalmopsylla* and *Paradoxopsyllus* are known as main or suspected vectors of plague, murine typhus, erysipeloid, listeriosis and salmonellosis in the Central Asia[107]. In an experiment it was showed that *L*. *segnis* is more successful in transmitting *R*. *typhi* to rats than *X*. *cheopis* [64]. *Leptopsylla aethiopica aethiopica* which transmits plague in Africa recently have been reported from Semnan province [50]; however its presence and identity in the region is very questionable.

People who travel to rural areas should consciously avoid flea bites especially in populations camping outside (herders, travelers, nomads) and avoid exposure to wild rodents and their fleas. In domestic areas, in order to prevent bites and thus disease transmission to humans, the floors and walls, as well as the rodents’ burrows around settlements, should theoretically be sprayed with insecticides. A few days later the application of rodenticides is necessary.

There were virtually no records of some flea species in a few provinces like North Khorasan (Fig 1). This is mainly due to inadequate inventories, especially in remote areas, or minorly due to the changing of geographical boundaries where the number of provinces in old classification has increased from 10 to 31 provinces.

In this paper we highlighted the geographical gaps on the Siphonaptera fauna of Iran. Generally, it shows that extensive fundamental and systematic research is still needed to determine the impact of off-host abiotic conditions and host identity (either mammal or bird) on host specificity, and on the potential for flea-borne diseases spread and transmission risk.

Co-evolution partly explains host-flea relationships which are translated into various degrees of host specificity (as shown in Tables 4–7) and morphological adaptations of the parasite [108]. Host specificity is important from the perspective of transmission of disease agents. It is more probable that, vertebrate hosts with related taxonomy or similar ecologies will have flea species that share similar pathogens. Depending on the level of infestation, flea species do not cause major problem to their hosts [108]. While some fleas species, virtually exclusively females, (*Echidnophaga* spp., *Vermipsylla* spp., *Dorcadia* spp., *Tunga* spp), spend much of their adult lives embedded or fixed in the host skin, this is far from being the rule. Indeed, most species jump on a host to feed intermittently before returning to the host dwelling place, usually a nest or burrow [6].

Den/nest making hosts (mammals or birds) display a more specific flea fauna than non roosting species [6]. It has been shown that fleas possibly appeared with mammals and speciated with rodents which still have the most speciose extant fauna (74%)[109].

Since rodent-borne, bat-borne and vector-borne diseases are the major rising concerns to health authorities, and threats to public health making inventories of the host and their ectopoarasitic fauna has become as never before a priority. Although most flea-borne diseases are not classified in the 17 neglected tropical diseases (NTDs) list made by the World Health Organization, this doesn’t mean those are unimportant or not causing an underestimated morbidity burden worldwide. The lack of recognition by major stakeholders, and the local lack of diagnostic tools and awareness are impeding improvements into flea-borne disease research. However, with about seven human or zoonotic highly pathogenic agents circulating among -possibly- the 117 flea species throughout Iran, there is an urgent need to organize and fund flea-host-pathogen ecological surveys in the face of rapid environmental and human behavioral changes.

### Conclusion

The first step in identifying the risk linked to flea exposure is to make a list of the species before any public health measures can be taken. Flea-borne diseases are caused by emerging and re-emerging infectious agents which distribution, prevalence and incidence are currently increasing. However, the data about fleas and their medical significance in different geographical regions of Iran is limited. We took the first step in this paper but supplementary studies are required to i) complete the list, especially in areas where there are no reportsor poor faunistic studies and ii) perform molecular screening of flea pools in order to detect specific pathogen circulation in domestic fauna and wildlife in order to prevent future epidemics.
